# Synergistic effects of silver ions and metformin against *enterococcus faecalis* under high-glucose conditions *in vitro*

**DOI:** 10.1186/s12866-021-02291-2

**Published:** 2021-09-29

**Authors:** Xuying Wu, Wei Fan, Bing Fan

**Affiliations:** The State Key Laboratory Breeding Base of Basic Science of Stomatology (Hubei-MOST) and Key Laboratory of Oral Biomedicine Ministry of Education, School and Hospital of Stomatology, Wuhan University, Wuhan, People’s Republic of China

**Keywords:** Diabetes, *Enterococcus faecalis*, Glucose, Metformin, Silver ion

## Abstract

**Background:**

This study aimed to evaluate the synergistic antibacterial activities of silver ions (Ag^+^) and metformin hydrochloride (Met) against *Enterococcus faecalis* (*E. faecalis*) under normal or high-glucose conditions.

**Results:**

The minimum inhibitory concentration, minimum bactericidal concentration, growth curves, and colony-forming units were used to evaluate the antibacterial effects of Ag^+^ and Met on planktonic *E. faecalis* in Brain Heart Infusion broth with or without additional glucose. The influences of Ag^+^ and Met on four weeks *E. faecalis* biofilm on human dentin slices was also tested. Cytotoxicity was tested on MC3T3-E1 osteoblastic cells using CCK-8 assays. The results indicated that *E. faecalis* showed higher resistance to drug treatment under high-glucose conditions. Ag^+^ (40 μg/mL) plus Met (3.2% or 6.4%) showed enhanced antibacterial activities against both planktonic *E. faecalis* and biofilm on dentin slices, with low cytotoxicity.

**Conclusions:**

Met enhanced the bactericidal effects of Ag^+^ against both planktonic and biofilm *E. faecalis* under normal or high-glucose conditions with low cytotoxicity. Further molecular studies are needed to be conducted to understand the mechanisms underlying the synergistic activity between Met and Ag^+^.

**Supplementary Information:**

The online version contains supplementary material available at 10.1186/s12866-021-02291-2.

## Background

Bacteria remaining in the root canal system after initial treatment is often responsible for endodontic treatment failure and refractory apical periodontitis (AP) [1]. These bacteria can survive and proliferate in the treated root canal by feeding on tissue fluid rich in glycoprotein from the periapical area and then induce or maintain inflammation around the periapical tissue [1]. Facultative anaerobic and gram-positive bacteria were reported to be the predominant flora in canals of treatment failure [2]. *Enterococcus faecalis* is one of the most frequently identified [3] due to its ability to survive in harsh environments such as extreme alkaline and nutrient deficiency conditions [4, 5]. In addition, antibiotics have been abused in recent decades with more and more serious resistance developed by many bacteria, and *E. faecalis* has also been reported to exhibit antibiotic resistance [6].

Diabetes mellitus (DM) is a metabolic disorder characterized by hyperglycemia, accompanied by immune dysfunction. Hyperglycemia leads to the formation of glycation end products, increasing tissue-oxidative stress and upregulating inflammatory responses, and reduces tissue repair capacity [7]. People with diabetes are particularly vulnerable to anaerobic bacteria or opportunistic infections because of limited collateral circulation [8]. *E. faecalis* was reported to have a higher detection rate (33%) in the infected root canals of patients with diabetes than in healthy patients (19%) [9]. Previous animal and human studies have suggested an association between AP and DM [10], manifested by a higher prevalence of AP, the larger size of the periapical lesions and bone destruction, delayed periapical healing, and a lower success rate of root canal therapy in patients with diabetes. Patients with long-term DM also showed a higher incidence of periradicular lesions than those with short-term DM [11]. DM must be considered critical factor affecting treatment prognosis [12].

Poorly controlled glycemia was significantly associated with AP, while metformin hydrochloride (Met), a typical medicine for hypoglycemia treatment, was associated with a lower prevalence of AP [13]. Recently, Wang et al. [14] reported that intracanal application of Met paste could reduce bone resorption associated with AP and promote bone defect healing. Local delivery of Met may be effective for controlling root canal infections. Met has been reported to be multifunctional as having anti-inflammation, anti-aging and anti-tumor properties [15–17]. Met has also shown antimicrobial properties against multiple pathogens, such as *Staphylococcus aureus* and *Pseudomonas aeruginosa*, and possess an adjuvant antimicrobial effect when combined with other antibiotics [18–20]; however, its effectiveness against *E. faecalis* remains unknown.

Ag^+^ is recognized as a broad-spectrum bactericidal metal ion widely used in medical applications; however, Ag^+^ has also been reported to cause discoloration, eye irritation, and allergic contact dermatitis [21]. *E. faecalis* has been proven to develop resistance against Ag^+^, leading to the metal’s decreased antibacterial effectiveness [22]. To solve these problems, Ag^+^ or nanoparticles has been used with other metal ions or chemicals to obtain a stronger synergistic antibacterial effect and lower cytotoxicity [23–26]. Despite this, the antibacterial ability of Ag^+^ against *E. faecalis* in diabetic environments, that is, high-glucose condition, remains unknown. In addition, whether there is a synergistic antibacterial effect against *E. faecalis* between Met and Ag^+^ under high-glucose conditions also remains unclear.

Based on these concerns, this study aimed to investigate the synergistic antibacterial effect of Met and Ag^+^ against *E. faecalis* under normal or high-glucose conditions in vitro. It was hypothesized that Ag^+^ plus Met would show a synergistic inhibitory effect on planktonic *E. faecalis* and biofilm on dentin slices either in normal or high glucose conditions.

## Results

### MIC and MBC determination

The minimum inhibitory concentration (MIC) and minimum bactericidal concentration (MBC) results of Met and AgNO_3_ with or without Met against *E. faecalis* in Brain Heart Infusion (BHI) broth with or without additional glucose were shown in Table 1. The MBC_99_ of Met was not detected at the tested concentrations. Under different concentrations of Met (3.2 and 6.4%), the MIC and MBC_99_ of AgNO_3_ decreased drastically.

**Table 1:** MIC and MBC of Met and AgNO_3_ with or without Met against *E. faecalis*

	BHI		BHIG	
	MIC	MBC	MIC	MBC
Met	12.80%	/	12.80%	/
AgNO_3_	160 μg/mL	320 μg/mL	160 μg/mL	640 μg/mL
AgNO_3_ + 3.2%Met	40 μg/mL	320 μg/mL	40 μg/mL	320 μg/mL
AgNO_3_ + 6.4%Met	10 μg/mL	40 μg/mL	20 μg/mL	80 μg/mL

*BHI* Brain Heart Infusion, *BHIG* Brain Heart Infusion broth with additional 25 mM glucose;

/: not detected at test concentrations

### Antibacterial effects against planktonic *E. faecalis*

**Fig. 1:**
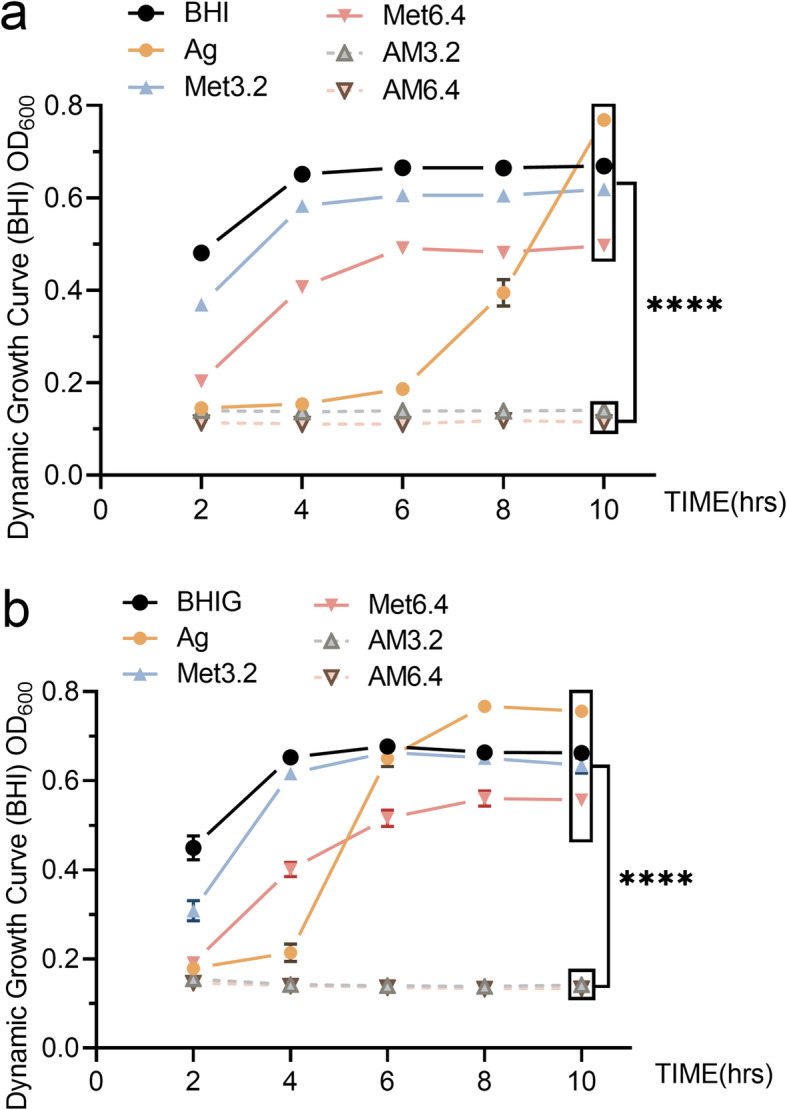
Dynamic growth curves of *E. faecalis* under different conditions**.** OD curves (OD_600_) of *E. faecalis* (1 × 10^8^ CFU/mL) incubated with Ag^+^ (40 μg/mL), Met (3.2 and 6.4%) alone or their combination or only culture media (n = 6). **A** in BHI broth; **B** in BHIG broth. ****: *P* < 0.0001(ANOVA)

**Fig. 2:**
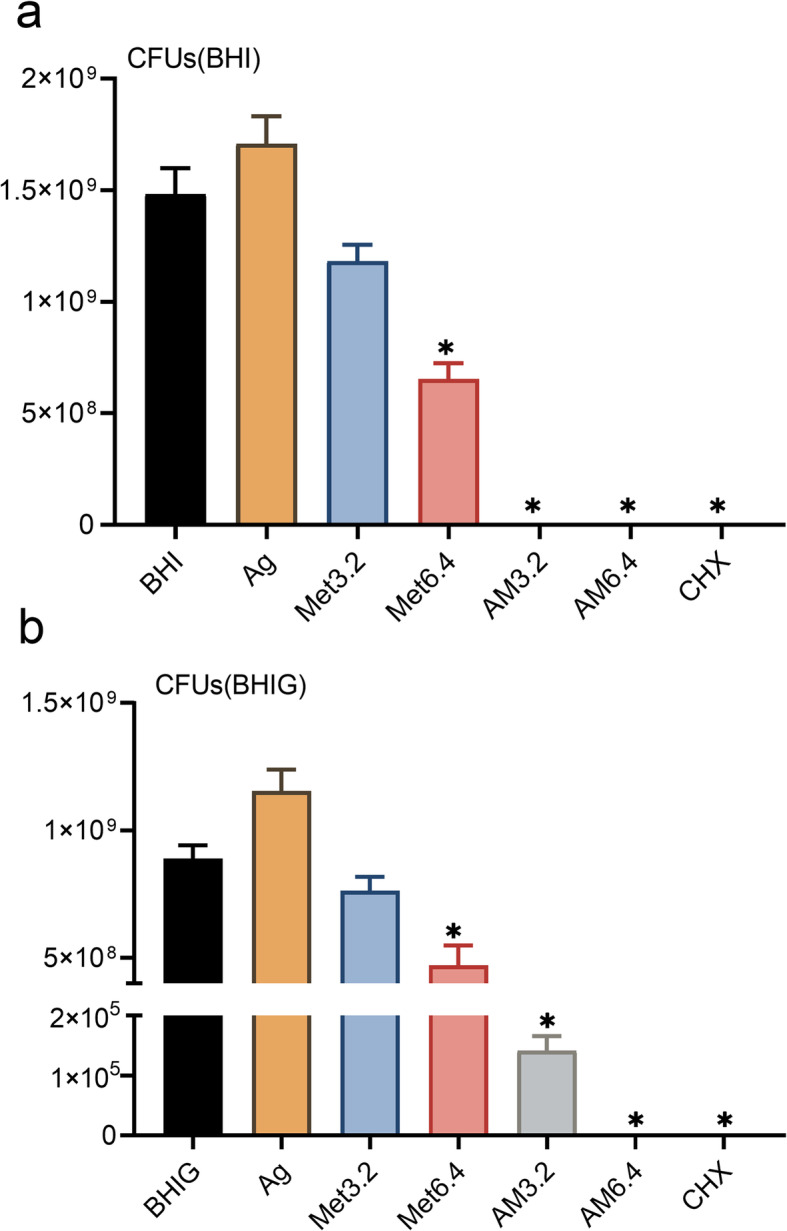
Antibacterial effects against planktonic *E. faecalis.* Comparisons of bacterial CFU counts among Ag, Met3.2, Met6.4, AM3.2, AM6.4, CHX and negative control groups (n = 6). **A** in BHI broth; **B** in BHIG broth. *:*P* < 0.05 (ANOVA) as compared to negative control group

After the test of MIC and MBC, dynamic growth curve tests and the colony-forming unit (CFU)-counting method were used to detect the antibacterial effects of Ag^+^ (40 μg/mL), Met (3.2 and 6.4%), and Ag^+^+Met on planktonic *E. faecalis* in BHI or BHIG broth. 2% chlorhexidine (CHX) was used as a positive control in the CFU-counting test. The results of dynamic growth curves in Fig. 1 indicate that the optical density (OD) values in the Ag^+^+Met group were significantly decreased (*p* < 0.0001) within 10 h. The CFU-counting tests (Fig. 2) showed that compared to Ag^+^ or Met alone, the Ag^+^+Met groups showed significantly lower CFU counts (*p* < 0.05) and increased antibacterial efficiencies (Table 2). Despite this, compared with normal BHI broth, the antibacterial efficiency decreased when more glucose was added to the culture medium.

**Table 2:** Antibacterial efficiencies (Mean ± S.E.M.) among groups using the CFU-counting method

%	Ag	Met3.2	Met6.4	AM3.2	AM6.4	CHX
BHI	−15.30 ± 8.33	20.25 ± 4.95	55.91 ± 4.82	100.00 ± 0.00	100.00 ± 0.00	100.00 ± 0.00
BHIG	−29.78 ± 9.41	14.23 ± 6.24	47.00 ± 8.74	99.98 ± 0.00	100.00 ± 0.00	100.00 ± 0.00

### Antibacterial effects against *E. faecalis* biofilm on dentin slices

**Fig. 3:**
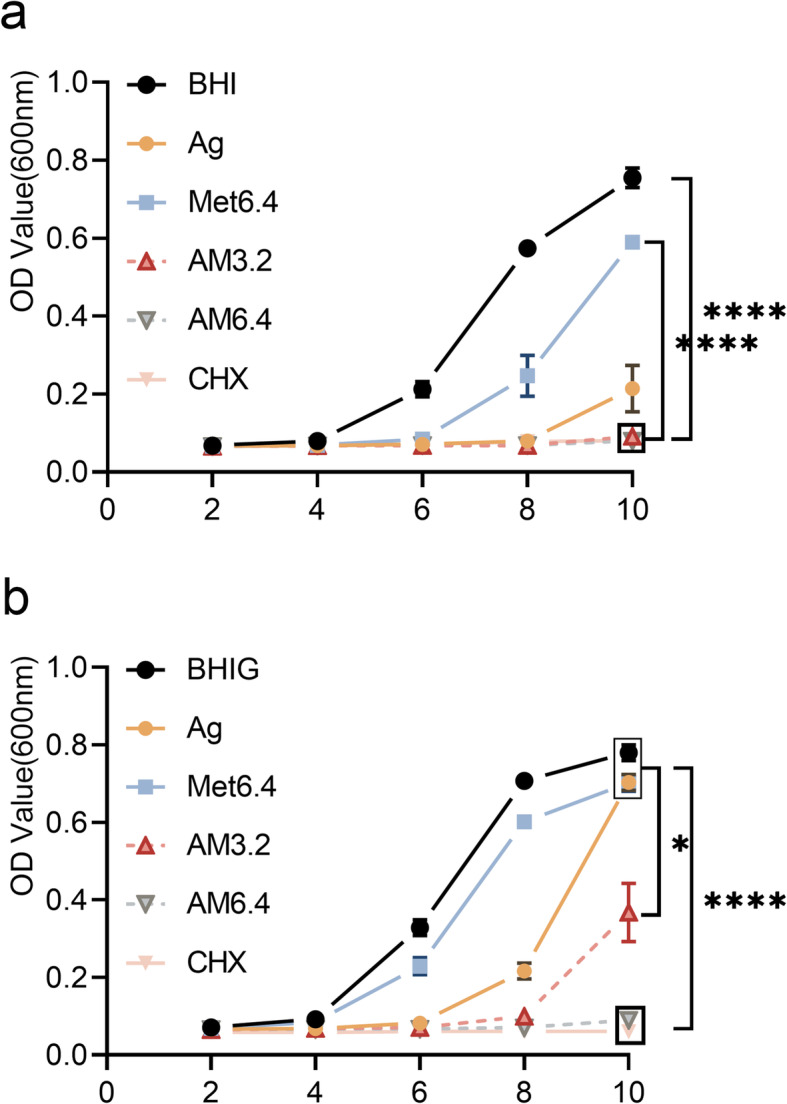
Antibacterial effect against *E. faecalis* biofilm grown on dentin slices. OD curves (OD_600_) of *E. faecalis* growth on dentin slices treated with Ag, Met6.4, AM3.2, AM6.4, CHX and PBS gel (n = 6) within 10 h. **A** in BHI broth. **B** in BHIG broth. *:*P* < 0.05(ANOVA). ****:*P* < 0.0001(ANOVA)

Four-week *E. faecalis* biofilm were cultured on human dentin slices, and their antibacterial effects were tested. The OD_600_ value measured within 10 h showed significantly decreased residual bacterial amount from the dentin slices in the Ag^+^+Met and CHX groups (Fig. 3, *P* < 0.05) compared to the control group, even under the high glucose condition.

### Cytotoxicity test

**Fig. 4:**
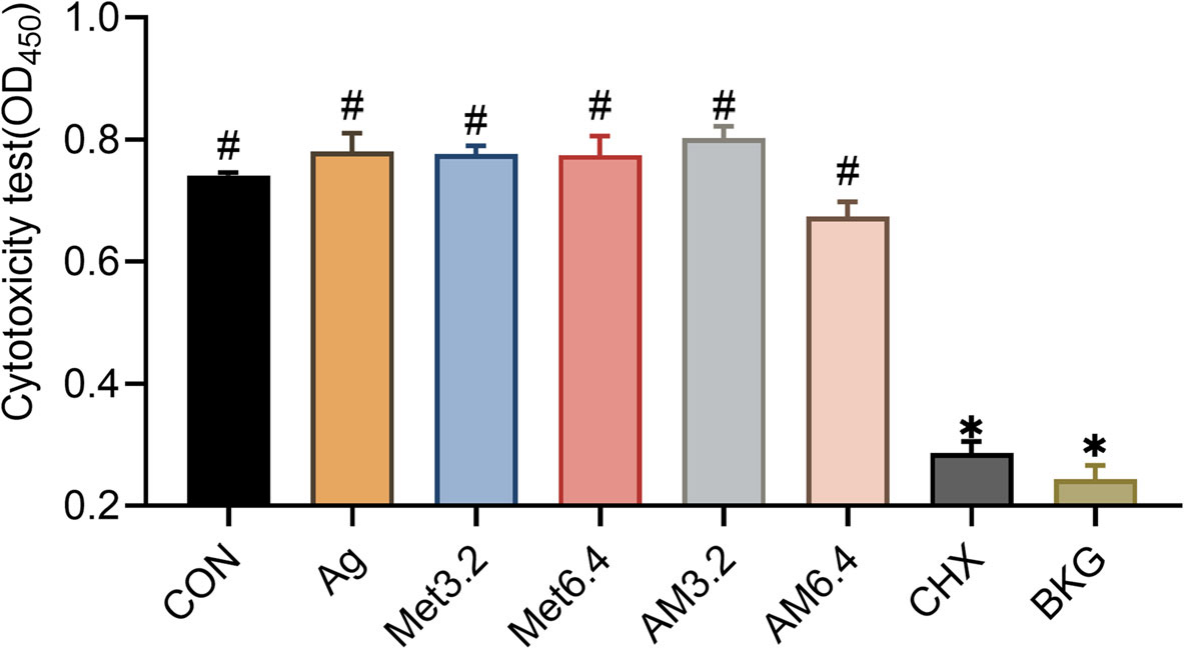
Cytotoxicity on MC3T3 -E1 cells using CCK-8 test. MC3T3-E1 cells (1 × 10^4^ cells) exposed to 10 μL Ag, Met3.2, Met6.4, AM3.2, AM6.4 and CHX solution (n = 6). **CON** cells cultured without treatment; **BKG** only CCK-8 medium background without cells or treatment. *:*P* < 0.05(ANOVA) as compared to CON group. #:*P* < 0.05(ANOVA) as compared to CHX group

The cytotoxicity of Ag^+^ and Met was assessed on MC3T3-E1 cells in vitro. The results of the CCK-8 test (Fig. 4) showed that the 2% CHX group had a significantly decreased OD_450_ value (*p* < 0.0001) and an inhibitory effect on cell proliferation. Although the AM6.4 group showed a slight inhibitory effect, there was no significant difference when compared with the negative control group (*p* > 0.05). All other groups showed low cytotoxicity (*p* > 0.05).

## Discussion

This study mainly focused on the synergistic inhibitory effect of Ag^+^ and Met on E. faecalis. MIC and MBC test, dynamic growth curve test and CFU-counting method were used to determine the synergistic antimicrobial effect on planktonic E. faecalis. The concentrations of metformin were selected based on the MIC test of metformin and pilot studies. The selected concentrations of 3.2 and 6.4% were 1/4 MIC and 1/2 MIC of metformin respectively, which could be better present the synergistic effect with silver ions than other concentrations as indicated in our pilot studies. According to the results, the combination of Ag^+^ (1/4 MIC) and Met (1/4 or 1/2 MIC) significantly inhibited the growth of planktonic *E. faecalis* in either normal or high glucose conditions. However, as previously reported, biofilm was more resistant to antimicrobial agents and harsh environments than planktonic bacteria [27]. Despite this, the application of Ag^+^ plus Met also showed strong synergistic inhibition effect on four-week *E. faecalis* biofilm on human dentin slices. Ag^+^ plus Met may be developed into a new effective and safe multifunctional antibacterial agent for root canal disinfection or other related medical areas under diabetic conditions.

Previous studies have demonstrated that high glucose media can promote *E. faecalis* biofilm formation [28, 29]. Another study suggested that when grown in the presence of glucose supplementation, the penetration of *E. faecalis* into dentinal tubules was deeper [30]. Local high-level glucose in diabetics may provide more energy resources for bacteria to survive starvation stress (a common condition for bacteria in filled root canals). In addition, human serum, as a nutrition source, favors *E. faecalis* to bind to collagen and invade dentinal tubules [31].

As a hypoglycemic medicine for the treatment of DM, Met has also been reported to be multifunctional as having anti-aging, anti-inflammation, anti-bacterial and anti-tumor properties [15–17, 19]. However, the potential application against *E. faecalis* has rarely been studied. In this study, MBC_99_ of Met was not detected at the tested concentrations, indicating the weak sterilization effect of Met on *E. faecalis*. In contrast, CHX, a biguanide compound, is widely used as an endodontic irrigant and medicament with confirmed activities against gram-positive and gram-negative bacteria, but showed lower cell viability compared to other irrigant in cytotoxicity tests [32, 33]. The differences of the bactericidal properties between CHX and Met may be owing to the biguanide configuration and the length of its alkyl side chain [34].

The molecular mechanism behind the antibacterial activity of Ag^+^ is thought to be the interaction between Ag^+^ and the bacterial cell envelope, DNA, enzymes and proteins (especially those with sulfhydryl groups) inside the cell, as well as the production of reactive oxygen species [35, 36]. Despite this, bacteria can develop resistance to Ag^+^ through various mechanisms such as the active efflux of ions from the cell [21]. The intrinsic efflux system of *E. faecalis* was also reported to be controlled by the energy derived from the catabolism of glucose and the proton concentration of the medium [37]. Proton motive force (PMF) is critical for membrane depolarization and the functions of the efflux pump, and the PMF can also be enhanced by glucose [38]. The antibacterial effect of Ag^+^ in high glucose environment could probably be influenced by the sufficient glucose as an energy source for the efflux system. Met disrupt the bacterial cytoplasmic membrane and undermine the function of the PMF-driven efflux pump, therefore to promote the intracellular accumulation of doxycycline and restore tetracyclines susceptibility to multidrug-resistant bacteria [20]. Met could possibly enhance the intracellular accumulation of Ag^+^ by increasing the permeability of the cell membrane and undermining the functions of the efflux pump, upon which the antibacterial effect of Ag^+^ was strengthened when acted together with Met.

Damage in the periapical tissues can be caused directly by virulence factors and production (such as lipoteichoic acids and extracellular superoxide production) of *E. faecalis*, or indirectly mediated by the host response [39, 40]. The nucleotide-binding domain, leucine-rich-containing family, pyrin domaincontaining-3 (NLRP3) inflammasome contributes to periapical inflammation caused by *E. faecalis*, and lipoteichoic acids was reported to induce NLRP3 inflammasome expression by activating the nuclear factor-kappa B (NF-κB) signaling pathway [41]. DM shows a higher susceptibility to anaerobic infection due to the severely hypoxic environment in inflamed lesions. DM can also influence periapical status by increasing cellular oxidant stress, inducing apoptosis of osteoblasts and inhibiting osteoblastic differentiation [42]. Metformin targets the NLRP3 inflammasome, and its anti-inflammatory effects are related to the NF-κB pathway and tumor necrosis factor alpha related genes [43]. In addition, studies have confirmed the therapeutic efficacy of intracanal Met medication for periapical lesions, such as suppressing inducible nitric oxide synthase (iNOS) and nitric oxide (NO) production and protecting osteoblasts against hypoxia-induced oxidative stress and apoptosis [43, 44]. Despite these functions, further molecular studies need to be conducted to understand the mechanism of the synergistic activity between Met and Ag^+^.

In conclusion, Ag^+^+Met synergism showed strong antibacterial activity against both planktonic and biofilm *E. faecalis* in either normal or high-glucose conditions with low cytotoxicity.

## Materials and methods

All procedures in this study were performed in accordance with relevant guidelines. *E. faecalis* ATCC 29212 (ATCC, Manassas, VA, USA) was used in this study and stored at −80 °C with 50% glycerol. For all experiments, bacteria were grown in Brain Heart Infusion (BHI, BD Biosciences, Bergen, New Jersey, USA) at 37 °C in an anaerobic incubator with 5% CO_2_ and 1% O_2_, and the *E. faecalis* bacterial density was standardized to the OD value of 1.0, using a spectrophotometer (UV-2401PC, Shimadzu Corporation, Japan) at a wavelength of 600 nm, corresponding to a cell density of 10^9^ CFU/mL. The standard bacterial suspension was used in subsequent experiments. BHIG broth was prepared by adding 25 mM glucose to the BHI broth.

### MIC and MBC determination

The MIC for AgNO_3_ and Met was determined using the microdilution method as previously described [25]. Briefly, AgNO_3_ or Met were prepared with sterile distilled water, and 100 μL of the test solution was added to a 96-well microtiter plate. Another 100 μL *E. faecalis* bacterial suspension (10^6^ CFU/mL) diluted in double-concentrated BHI or BHIG broth was transferred into each well. For AgNO_3_ or Met alone, the final concentrations ranged from 5 to 640 μg/mL or from 0.1 to 12.8%, respectively. For the AgNO_3_ + Met test, the concentration of Met remained unchanged (3.2% or 6.4%) with AgNO_3_ ranging from 5 to 640 μg/mL. the plates were incubated for 24 h at 37 °C in an anaerobic incubator with 5% CO_2_ and 1% O_2_ in the dark. A micro-plate reader (Power Wave XS2, BioTek Instruments, VT, USA) was used at 600 nm to evaluate cell growth. Each assay contained both experimental and negative controls (media only). The MIC was defined as the lowest concentration that inhibited *E. faecalis* growth after incubation, showing OD_600_ values closest to hose of the negative controls. For MBC experiments, 100 μL of solutions from wells without turbidity were cultured on BHI agar. At least three wells (MIC, MIC × 2 and MIC × 4) were tested. MBC_99_ was determined to be the concentration with at least 99% inhibition. The measurements were performed in triplicate.

### Dynamic growth curve determination

The antimicrobial effect identified of Ag^+^ and Met solutions was identified according to previous studies [22]. Briefly, fresh *E. faecalis* suspension was adjusted to 10^8^ CFU/mL and incubated with AgNO_3_ solutions (40 μg/mL), Met solutions (3.2% or 6.4%) or their combinations in BHI and BHIG broth at 37 °C in an anaerobic incubator with 5% CO_2_ and 1% O_2_ in the dark. Aliquots were retrieved and optical density was measured at 600 nm every 2 h. The negative control was defined as the untreated *E. faecalis*. Each test was repeated six times.

### Colony forming units (CFU)

The CFU counting method [45] was used to detect the antibacterial effects of Ag^+^ and Met. Briefly, 1 mL suspension (10^4^ CFU/mL) of *E. faecalis* was incubated with different concentrations of AgNO_3_ and Met in BHI and BHIG broth for 24 h at 37 °C in an anaerobic incubator with 5% CO_2_ and 1% O_2_ in the dark. 2% CHX was used as a positive control because of its confirmed bactericidal effectiveness. After serial 10-fold dilution or no, 10 μL of bacterial sample from each group was retrieved and inoculated on BHI agar plates for a further 24 h. the CFU of *E. faecalis* was counted to evaluate the number of viable bacteria. The test was repeated six times for each group.

According to the results of the CFU counting test, the antibacterial efficiency (%) against *E. faecalis* was calculated as:

$$ \mathrm{Antibacterial}\ \mathrm{efficiency}\ \left(\%\right)=\frac{\mathrm{CFU}\ \mathrm{of}\ \mathrm{negative}\ \mathrm{control}\ \mathrm{groups}-\mathrm{CFU}\ \mathrm{of}\ \mathrm{treatment}\ \mathrm{groups}}{\mathrm{CFU}\ \mathrm{of}\ \mathrm{negative}\ \mathrm{control}\ \mathrm{groups}\ } $$

### Antibacterial effects against *E. faecalis* biofilm on dentin slices

Extracted third molar teeth were collected under the approval (2017–11) of the Ethics Committee of the School and Hospital of Stomatology, Wuhan University and cut into dentin slices with a size of 4 mm (width) × 4 mm (length) × 1 mm (thickness). All slices were thoroughly cleaned by ultrasound using distilled water, 5.25% sodium hypochlorite and 17% ethylenediaminetetraacetic acid in sequence for 4 min each, with distilled water for 1 min as the final wash. Finally, they were autoclaved at 121 °C in distilled water for 20 min [26, 46].

For the antibacterial effect test, all dentin slices were evenly divided into two groups and soaked in 1 mL *E. faecalis* suspension (10^8^CFU/mL) in BHI or BHIG broth at 37 °C in an anaerobic incubator with 5% CO_2_ and 1% O_2_ for four weeks [47, 48] Fresh BHI or BHIG broth was changed every 48 h. For each culture medium, dentin slices were washed with phosphate-buffered saline (PBS) and randomly divided into six groups (n = 6), and then embedded with AgNO_3_, Met, AgNO_3_-Met, and CHX gels for 7 days at 37 °C. Gels were prepared by mixing 0.15 g methylcellulose (Aladdin Industrial Corporation, Shanghai, China) with 2 mL of tested solutions. After 7 days, the slices were gently washed with PBS for three times and then soaked in 2 mL of fresh BHI or BHIG broth. Biofilm cells were dispersed by vortex mixing for 1 min. During 10 h of incubation, 200 μL suspension was retrieved for absorbance measurement at 600 nm every 2 h. Dentin slices without medication (methylcellulose gel only) were set as the negative control, while the group treated with 2%CHX gel was set as the positive control.

### Cytotoxicity assays

The cytotoxicity of Ag^+^ and Met on MC3T3-E1 subclone 14 osteoblastic cells (ATCC CRL-2594) in vitro was assessed using the cell counting kit-8 (CCK-8, Dojindo Laboratories, Kumamato, Japan) according to the previous studies [49]. Two hundred microliters of MC3T3-E1 cells (1 × 10^4^ cells) were seeded into each well of a 96-well plate and cultured at 37 °C in a 5% CO_2_ atmosphere in α-minimum essential medium (α-MEM; Hyclone, Logan, UT, USA) with 10% fetal bovine serum (Hyclone) and 1% penicillin/streptomycin (Hyclone). The tested solutions were sterilized using a 0.22-μm filter (Merck Millipore Ltd., Darmstadt, Germany). After incubation for 24 h, the culture medium was replaced with 200 μL of α-MEM and 10 μL of different dilutions containing AgNO_3_(40 μg/ mL), Met (3.2% or 6.4%) or their combinations and CHX (2%). After incubating for another 24 h, the supernatant was removed and the plate was washed with PBS. Finally, 100 μL of fresh α-MEM and 10 μL of CCK-8 were added and incubated in the dark for 1 h. The supernatant was transferred to a new 96-well plate, and the absorbance at 450 nm was measured using a microplate reader. The untreated group was used as the control. Wells containing only CCK-8 and α-MEM were used for background recordings (BKG). Each group included six repeated wells.

### Statistical analysis

All data are expressed as the mean ± S.E.M.. Data analysis was performed using the Graphpad Prism 8 (San Diego,CA). One-way analysis of variance (ANOVA) with a post hoc Dunnett t-test was performed for CFU and cytotoxicity assays. A two-way ANOVA was performed for the dynamic growth curve and biofilm assays. Statistical significance was set at *P* < 0.05.

## Supplementary Information

**Additional file 1: Table S1.** Data of dynamic growth curve test were shown as mean OD_600_ and Standard Error of Means (SEM), which was plotted in Fig. 1. **Table S2.** Data of colony-forming units (CFU)-counting test were shown as mean counting and SEM, which was plotted in Fig. 2. **Table S3.** Data of biofilm test on dentin slices were shown as mean OD_600_ and SEM, which was plotted in Fig. 3. **Table S4.** Data of cytotoxicity assays were shown as mean OD_450_ and SEM, which was plotted in Fig. 4.

## Data Availability

All data generated or analyzed during this study are included in this published article and its supplementary information files.
