# Supplementary material for: Synergistic effects of silver ions and metformin against enterococcus faecalis under high-glucose conditions in vitro
Source: BMC Microbiol. 2021 Sep 29;21:261. doi: 10.1186/s12866-021-02291-2 (PMC8482635; doi:10.1186/s12866-021-02291-2)
Supplement: Supplementary file 1 — Additional file 1: Table S1. Data of dynamic growth curve test were shown as mean OD600 and Standard Error of Means (SEM), which was plotted in Fig. 1. Table S2. Data of colony-forming units (CFU)-counting test were shown as mean counting and SEM, which was plotted in Fig. 2. Table S3. Data of biofilm test on dentin slices were shown as mean OD600 and SEM, which was plotted in Fig. 3. Table S4. Data of cytotoxicity assays were shown as mean OD450 and SEM, which was plotted in Fig. 4. [file 12866_2021_2291_MOESM1_ESM.pdf]

Synergistic effects of silver ions and metformin against *Enterococcus faecalis* under high-glucose conditions *in vitro*

Xuying Wu[a], Wei Fan [a,b], Bing Fan[a,b]

[a]: The State Key Laboratory Breeding Base of Basic Science of Stomatology (Hubei-MOST) and Key Laboratory of Oral Biomedicine Ministry of Education, School and Hospital of Stomatology, Wuhan University, Wuhan, People's Republic of China

[b]: Corresponding authors: Wei Fan (weifan@whu.edu.cn) & Bing Fan (bingfan@whu.edu.cn)

Supplementary Information

Table S1. Data of dynamic growth curve test were shown as mean OD_{600} and Standard Error of Means (SEM), which was plotted in Figure 1.

		2h		4h		6h		8h		10h	
		Mean	SEM	Mean	SEM	Mean	SEM	Mean	SEM	Mean	SEM
BHI	Ag	0.145	0.003	0.154	0.002	0.186	0.004	0.394	0.029	0.769	0.013
	Met3.2	0.369	0.009	0.582	0.007	0.606	0.010	0.605	0.010	0.618	0.011
	Met6.4	0.203	0.006	0.407	0.005	0.491	0.006	0.482	0.005	0.497	0.007
	AM3.2	0.139	0.005	0.137	0.006	0.139	0.005	0.138	0.006	0.140	0.004
	AM6.4	0.114	0.002	0.111	0.001	0.111	0.001	0.119	0.005	0.114	0.002
	BHI	0.481	0.008	0.652	0.009	0.665	0.009	0.665	0.006	0.669	0.005
BHIG	Ag	0.179	0.003	0.214	0.020	0.650	0.019	0.767	0.012	0.756	0.010
	Met3.2	0.308	0.022	0.616	0.006	0.664	0.004	0.651	0.009	0.634	0.017
	Met6.4	0.191	0.009	0.401	0.016	0.516	0.019	0.560	0.017	0.556	0.012
	AM3.2	0.155	0.006	0.142	0.002	0.139	0.001	0.138	0.001	0.141	0.002
	AM6.4	0.147	0.005	0.140	0.003	0.136	0.003	0.133	0.002	0.134	0.002
	BHIG	0.449	0.027	0.653	0.005	0.677	0.014	0.664	0.010	0.662	0.011

Table S2. Data of colony-forming units (CFU)-counting test were shown as mean counting and SEM, which was plotted in Figure 2.

	BHI		BHIG	
	Mean	SEM	Mean	SEM
CON	1.48×10^{9}	0.12×10^{9}	8.90×10^{8}	0.52×10^{8}
Ag	1.71×10^{9}	0.12×10^{9}	1.16×10^{9}	0.08×10^{9}
Met3.2	1.18×10^{9}	0.07×10^{9}	7.63×10^{8}	0.56×10^{8}
Met6.4	6.53×10^{8}	0.71×10^{8}	4.72×10^{8}	0.78×10^{8}
AM3.2	0	0	1.42×10^{5}	0.24×10^{5}
AM6.4	0	0	0	0
CHX	0	0	0	0

Table S3. Data of biofilm test on dentin slices were shown as mean OD_{600} and SEM, which was plotted in Figure 3.

		2h		4h		6h		8h		10h	
		Mean	SEM	Mean	SEM	Mean	SEM	Mean	SEM	Mean	SEM
BHI	BHI	0.068	0.001	0.080	0.002	0.213	0.019	0.574	0.016	0.755	0.025
	Ag	0.067	0.000	0.068	0.000	0.071	0.002	0.079	0.004	0.214	0.059
	Met6.4	0.067	0.001	0.069	0.001	0.084	0.005	0.247	0.053	0.590	0.013
	AM3.2	0.066	0.000	0.067	0.000	0.068	0.000	0.068	0.000	0.091	0.004
	AM6.4	0.066	0.001	0.068	0.000	0.068	0.000	0.068	0.001	0.079	0.003
	CHX	0.065	0.000	0.067	0.001	0.070	0.003	0.078	0.007	0.080	0.008
BHIG	BHIG	0.071	0.001	0.092	0.003	0.328	0.021	0.707	0.014	0.780	0.021
	Ag	0.066	0.001	0.068	0.001	0.082	0.002	0.217	0.021	0.703	0.020
	Met6.4	0.069	0.001	0.085	0.002	0.229	0.022	0.601	0.013	0.701	0.021
	AM3.2	0.065	0.000	0.068	0.001	0.070	0.001	0.099	0.010	0.368	0.075
	AM6.4	0.066	0.000	0.066	0.000	0.067	0.000	0.070	0.001	0.089	0.007
	CHX	0.059	0.004	0.057	0.005	0.060	0.005	0.060	0.005	0.060	0.005

Table S4. Data of cytotoxicity assays were shown as mean OD_{450} and SEM, which was plotted in Figure 4.

	Ag	Met3.2	Met6.4	AM3.2	AM6.4	CON	BKG	CHX
Mean	0.781	0.777	0.775	0.803	0.675	0.742	0.244	0.287
SEM	0.030	0.014	0.031	0.020	0.024	0.005	0.023	0.019
